# Efficacy of Mitochondrial Antioxidant Plastoquinonyl-decyl-triphenylphosphonium Bromide (SkQ1) in the Rat Model of Autoimmune Arthritis

**DOI:** 10.1155/2016/8703645

**Published:** 2016-05-18

**Authors:** Alexander A. Andreev-Andrievskiy, Nataliya G. Kolosova, Natalia A. Stefanova, Maxim V. Lovat, Maxim V. Egorov, Vasily N. Manskikh, Roman A. Zinovkin, Ivan I. Galkin, Anastasia S. Prikhodko, Maxim V. Skulachev, Alexander N. Lukashev

**Affiliations:** ^1^Institute of Mitoengineering, Lomonosov Moscow State University, Moscow, Russia; ^2^Faculty of Biology, Lomonosov Moscow State University, Moscow, Russia; ^3^Institute of Cytology and Genetics, Novosibirsk, Russia; ^4^Belozersky Institute of Physico-Chemical Biology, Lomonosov Moscow State University, Moscow, Russia; ^5^Martsinovsky Institute of Medical Parasitology and Tropical Medicine, Sechenov First Moscow State Medical University, Moscow, Russia

## Abstract

Rheumatoid arthritis is one of the most common autoimmune diseases. Many antioxidants have been tested in arthritis, but their efficacy was, at best, marginal. In this study, a novel mitochondria-targeted antioxidant, plastoquinonyl-decyl-triphenylphosphonium bromide (SkQ1), was tested* in vivo* to prevent and cure experimental autoimmune arthritis. In conventional Wistar rats, SkQ1 completely prevented the development of clinical signs of arthritis if administered with food before induction. Further, SkQ1 significantly reduced the fraction of animals that developed clinical signs of arthritis and severity of pathological lesions if administration began immediately after induction of arthritis or at the onset of first symptoms (day 14 after induction). In specific pathogen-free Wistar rats, SkQ1 administered via gavage after induction of arthritis did not reduce the fraction of animals with arthritis but decreased the severity of lesions upon pathology examination in a dose-dependent manner. Efficacious doses of SkQ1 were in the range of 0.25–1.25 nmol/kg/day (0.13–0.7 *μ*g/kg/day), which is much lower than doses commonly used for conventional antioxidants. SkQ1 promoted apoptosis of neutrophils* in vitro*, which may be one of the mechanisms underlying its pharmacological activity. Considering its low toxicity and the wide therapeutic window, SkQ1 may be a valuable additional therapy for rheumatoid arthritis.

## 1. Introduction

Rheumatoid arthritis (RA) is one of the most prevalent autoimmune diseases. Infection by Epstein-Barr virus, human herpesvirus 6, and polyetiological periodontitis has been named among factors that could promote disease development and progression [1–3]. Commonly used clinical regimens can slow the disease progression; however, they have significant side effects and cannot be administered indefinitely. Immune therapies have a huge potential [4], but they are currently expensive due to high manufacturing costs of biopharmaceuticals. In addition, their side effects and potential for prolonged use are not entirely clear. Development of drugs to treat arthritis has mainly been aimed at protein interactions that are relatively well understood. Another component of inflammation signaling, the reactive oxygen species (ROS), has largely been neglected because it is very impractical to study molecules that have a short decay time and are not detectable by immunological assays, although there was a solid theoretical background for such therapies (reviewed in [5]). Empirical trials of antioxidants, such as hydrogen [6], melatonin [7], resveratrol [8], vitamins, and herbal extracts [9, 10], provided evidence of efficacy in animal models of RA. However, these results were hard to convert into clinical practice because the high doses of conventional antioxidants that are required to achieve efficacy can produce side effects.

**Figure 1:**
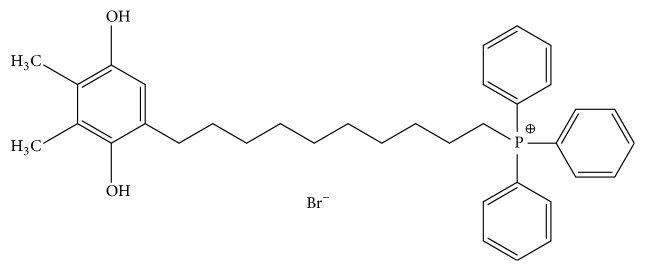
Structural formula of SkQ1 (plastoquinonyl-decyl-triphenylphosphonium bromide).

Plastoquinonyl-decyl-triphenylphosphonium (SkQ1; Figure 1) is a fusion of plastoquinone, a plant analog of ubiquinone, and triphenylphosphonium, a lipophilic cation that has a positive charge smeared over three aromatic rings. The latter part of the molecule acts as a transmembrane transporter to the negatively charged inner lumen of the mitochondria [11], where the plastoquinone part provides rechargeable ROS-scavenging activity. This antioxidant activity was demonstrated in isolated mitochondria, in cell culture [12], and* in vivo* in laboratory animals [13]. Later studies showed that SkQ1 is especially efficient in protection of mitochondrial lipid cardiolipin from oxidation [14, 15]. The concentration of SkQ1 molecules in the mitochondria is estimated to be up to 8 logs higher than that in the cell culture medium [12]. Pharmacological efficacy of SkQ1 was demonstrated in a number of animal models that are associated with ROS-mediated damage, such as ischemia-reperfusion, autoimmune inflammation, and senile disorders (reviewed in [16]).

Previous studies suggested that SkQ1 potentially has a very wide therapeutic window (difference between beneficial antioxidant and toxic prooxidant concentrations) and might exhibit therapeutic efficacy at much lower doses than conventional antioxidants [12]. This study was aimed to test efficacy of a mitochondria-targeted antioxidant SkQ1 in the rat model of autoimmune arthritis.

## 2. Materials and Methods

### 2.1. Compound

Plastoquinonyl-decyl-triphenylphosphonium bromide (SkQ1) was synthesized as described previously [12]. In Series I (see below), an oxidized form of the compound (Figure 1) was used. SkQ1 was dissolved in ethanol to 10 mg/mL and then in water to a working concentration. In Series II, a reduced form of SkQ1 (a candidate drug formulation) was used. Reduction of SkQ1 was provided by addition of ascorbic acid (SkQ1 to ascorbic acid ratio 2.5 : 97.5).

### 2.2. *In Vivo* Experiments

Animal experiments were carried out at two laboratories. As experimental conditions differed significantly between these experiments, they are referred to as Series I (Institute of Cytology and Genetics (ICG), Siberian Division of the Russian Academy of Sciences, Novosibirsk) and Series II (Institute of Mitoengineering, Moscow State University, Moscow).

Series I was carried out using conventional in-house bred Wistar rats, which were obtained from the Shared Center for Genetic Resources of Laboratory Animals of the ICG. At the age of 4 weeks, the pups were weaned, housed in groups of five animals per cage, and kept under standard laboratory conditions (22°C ± 2°C, 60% relative humidity, and natural lighting). The animals were provided with standard rodent diet (PK-120-1, Laboratorsnab Ltd., Russia) and water* ad libitum.* Experimental protocols were approved by the ethical committee of the Institute of Cytology and Genetics.

Arthritis was induces by subcutaneous (s.c.) injection of 250 *μ*g of type 2 chicken collagen (Sigma-Aldrich, St. Louis, USA) dissolved in a 1 : 1 mixture of incomplete and complete Freund's adjuvant (Sigma-Aldrich) to a total volume of 250 *μ*L. In experiment I-1 (preventive administration), SkQ1 (250 nmol/kg/day) was applied with a dispenser on pieces of cottage cheese that were individually fed to experimental animals starting 55 days before induction of arthritis and until the end of experiment. Control rats received untreated cottage cheese. In this experiment, there were groups of rats aged 4 months (“young animals”) and 20 months (“old animals”). The number of rats in the mock-immunized, immunized-untreated, and immunized-treated groups was 10, 10, and 10 (young rats) and 10, 8, and 10 (old rats), respectively. Severity of arthritis was evaluated by clinical scoring [17] at days 15, 18, 20, 25, 28, and 30. Rats were sacrificed at day 32 after arthritis induction.

In experiment I-2 (treatment), ten 3-month-old Wistar rats were mock-immunized (negative control). An untreated control group (*n* = 15) and two experimental groups (*n* = 15 in each) were immunized with chicken collagen as described above. SkQ1 was administered into the oral cavity of rats with a dispenser (250 nmol/kg/day) beginning at day 1 or day 14 after arthritis induction. Vehicle solution (2% ethanol in water) was administered to control rats. Animals were examined at days 14, 17, 19, 21, 24, 26, 28, and 30 after arthritis induction. Rats were sacrificed at day 32 after arthritis induction.

Series II was carried out in the specific pathogen-free (SPF) animal house of the Institute of Mitoengineering, Moscow. The experiment was approved by the Bioethics committee of the Institute of Mitoengineering (Protocol number 19, May 10, 2011). SPF Wistar rats aged 5-6 weeks were obtained from the Pushchino Breeding Facility (Moscow region, Russia). Autoimmune arthritis was induced by immunization with 250 *μ*g type 2 porcine collagen (Chondrex, USA) in complete Freund's adjuvant according to the published protocol [18]. SkQ1 was administered via oral gavage at 50, 250, and 1250 nmol/kg/day. Control rats received vehicle (ascorbic acid solution without SkQ1). In a preliminary experiment the vehicle solution did not affect clinical course of RA (data not shown). Rats were sacrificed at day 30 after arthritis induction.

Joint specimens were fixed in 10% buffered formalin solution (pH = 7,4), decalcified in 14% ethylenediaminetetraacetate solution, and paraffin-embedded. Horizontal (paw) and vertical (knee) microtome sections (4 *μ*m) were performed. Histopathological examination of the knee (Series I) and hind paw (Series II) joints was performed upon staining with hematoxylin and eosin. Acidic glycosaminoglycans were visualized with alcian blue staining. Distribution of collagen was visualized by Mallory's staining. All examinations and evaluations were done on blinded slides by professional animal pathologist. Five high-power magnification fields (HMF) were scored for each animal based on the criteria published in [19, 20]. Synovial inflammation was scored based on the amount of infiltrating mononuclear cells as follows: 0, absent; 1, mild (1–10%); 2, moderate (11–50%); 3, severe (51–100%). Synovial hyperplasia was scored as 0, absent; 1, mild (3-4 layers for knee and 2 layers for paw); 2, moderate (5-6 layers for knee and 3 and more layers for paw); 3, severe (more than 6 layers for knee and 3 layers for paw). Cartilage erosion was evaluated based on the fraction of the cartilage surface that was eroded: 0, absent; 1, mild (1–10%); 2, moderate (10–30%); 3, severe (more than 30%). Bone erosion was scored as 0, none; 1, minor erosion(s) observed only at HMF; 2, moderate erosion(s) observed at low magnification; 3, severe transcortical or subtranscortical erosion(s).

### 2.3. Neutrophil Isolation

All experimental procedures were reviewed and approved by the Institutional Ethics Committee of the A. N. Belozersky Institute of Lomonosov Moscow State University before the study began. Written informed consent was obtained from blood donors. Peripheral blood was collected from healthy human donors into heparin-containing tubes. Neutrophils were isolated by dextran sedimentation and centrifugation on a Ficoll-Paque gradient. Residual erythrocytes were lysed by treating the cell pellets with distilled water for 45 seconds. The collected neutrophils were resuspended in RPMI-1640 medium (Paneco, Russia) containing 10% low-endotoxin fetal calf serum (PAA Laboratories, Germany). Isolated neutrophils were consistently >98% pure by modified Wright-Giemsa staining and >98% viable as determined by trypan blue dye exclusion.

### 2.4. Assessment of Neutrophil Apoptosis

Purified neutrophils (1 × 10^6^/mL) were treated with SkQ1 and/or with mitochondrial debris and incubated in a final volume of 0.5 mL for 22 hours at 37°C in a 5% CO_2_ humidified incubator. Neutrophil survival was assessed after 22 h using annexin V staining. Briefly, cells were washed twice with PBS, incubated for 20 min in the dark at 37°C with annexin-V-fluorescein isothiocyanate and propidium iodide, and analyzed with a Beckman Coulter FC500 system as described in [21]. Neutrophil apoptosis is expressed as the percentage of annexin-V-positive and propidium-iodide-negative cells.

### 2.5. Preparation of Mitochondrial Debris (MTD)

Mitochondria were isolated from rat liver or from the human endothelial cell line Ea.hy926. All procedures were performed at 4°C. Cells were disrupted using a Potter homogenizer in buffer containing 10 mM Tris-HCl, pH 7.5, 2 mM EDTA, 50 mM NaCl, and 0.2 M sucrose. Cell debris was discarded by centrifugation at 1000 ×g for 10 min. The supernatant was centrifuged at 10,000 ×g for 20 min and the mitochondrial pellet was obtained. Mitochondria were resuspended in PBS and disrupted by ultrasound sonication (eight times for 20 s with 40 s intervals). Mitochondrial membranes were removed by centrifugation at 100,000 ×g for 50 min, and supernatant containing soluble MTD was obtained and stored in aliquots at −70°C. Protein concentration of the MTD solution was determined by the Bradford method using the Bio-Rad protein assay. For the neutrophil survival studies, the final protein concentration of MTD was 2.5 mg/mL.

### 2.6. Statistical Analysis

Statistical analysis was performed using GraphPad Prizm 5.0. Survival was analyzed with Fisher's exact test and log-rank test. Histopathological scores were analyzed with Kruskal-Wallis test with Dunn's postcorrection. Neutrophil survival was assayed using ANOVA with Dunn's postcorrection.

## 3. Results 

### 3.1. Experiment I-1

Immunization of young Wistar rats with collagen resulted in development of arthritis in 7/10 animals. The disease was progradient (without spontaneous remissions) and progressed to clinical grade 4 in six animals. Preventive administration of SkQ1 with food completely averted development of arthritis in young Wistar rats (*p* < 0.01, Fisher's exact test; *p* < 0.01, log-rank test). In old animals, 6/8 rats developed clinical signs of arthritis by day 18. However, the disease resolved in three animals by day 25, and, therefore, it was not appropriate to analyze disease-free progression in this experiment. Pretreatment with SkQ1 resulted in a decrease in the proportion of animals (3/10) that had clinical signs of arthritis at one or more observation points and a decrease in the proportion of animals (2/20) that had arthritis at the experiment endpoint (day 25). However, these results did not differ significantly from the untreated control group (*p* > 0.05, Fisher's exact test).

**Figure 2:**
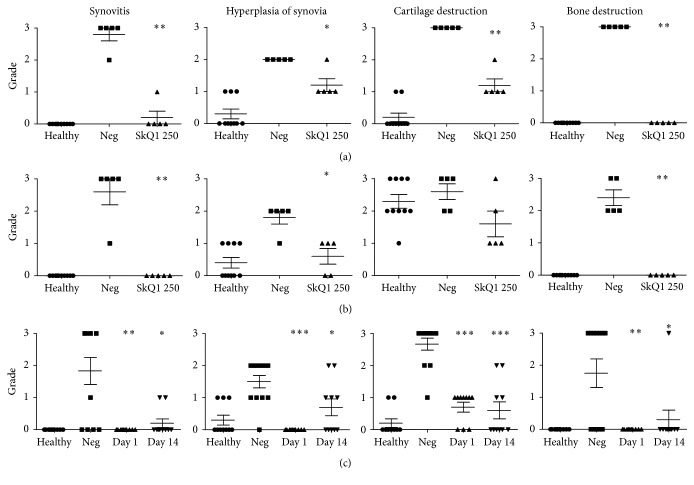
Pathological findings in 4-month-old (a) and 20-month-old (b) Wistar rats that were pretreated for 55 days with SkQ1 (250 nmol/kg/day) prior to induction of autoimmune arthritis and in 4-week-old rats treated with the same dose of SkQ1 daily from day 1 or day 14 after disease induction (c). Animals were sacrificed 32 days after arthritis induction. Severity of indicated pathological signs of arthritis was assayed upon H&E staining. Acidic glycosaminoglycans (cartilage) were visualized by alcian blue staining. Distribution of collagen was visualized by Mallory's staining. All evaluations were done on blinded slides. Five high magnification fields were assayed per sample. Severity of pathological manifestations was scored by 3-point scale, from 0 for normal tissue to 3 for maximum damage. Statistical significance of difference between the negative control and pretreated groups was tested with the Kruskal-Wallis test; ^*∗*^ *p* < 0.05; ^*∗∗*^ *p* < 0.01; ^*∗∗∗*^ *p* < 0.001.

Pathological investigation of the knee joint of five randomly selected rats per group showed that all untreated rats developed clear signs of arthritis (Figure 2(a)), which were significantly less pronounced or completely absent (bone destruction) in rats pretreated with SkQ1. Similar observations could be seen in old rats (Figure 2(b)); however, as the animals at 20 months already had age-related cartilage degeneration, autoimmune arthritis did not produce additional damage to the cartilage. It is of note that the degree of cartilage degeneration in old rats was lower in the SkQ1-treated group than in the untreated animals (not statistically significant).

### 3.2. Experiment I-2

**Figure 3:**
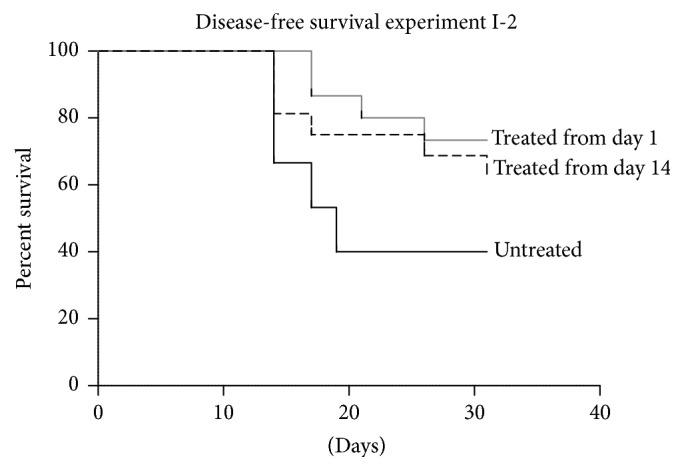
Disease-free survival of 3-month-old Wistar rats upon induction of autoimmune arthritis and treatment with 250 nmol/kg/day of SkQ1 initiated at day 1 or day 14 after arthritis induction. Day 0: induction of arthritis.

Treatment of experimental autoimmune arthritis in young rats resulted in delayed progress of arthritis and a reduction in the number of animals that developed clinical signs of arthritis (Figure 3). The effect of treatment that commenced at day 1 after arthritis induction on disease-free survival was statistically significant (*p* < 0.05, log-rank test). Moreover, only one animal in each treated group progressed to severe arthritis (grade 4 by clinical scale [22]) compared to three animals in the control group; however, this effect was not statistically significant (data not shown). Pathological examination (Figure 2(c)) revealed a significant reduction or abrogation of inflammatory and degenerative lesions, which was more pronounced in the group treated from day 1 after arthritis induction.

### 3.3. Experiment II

**Figure 4:**
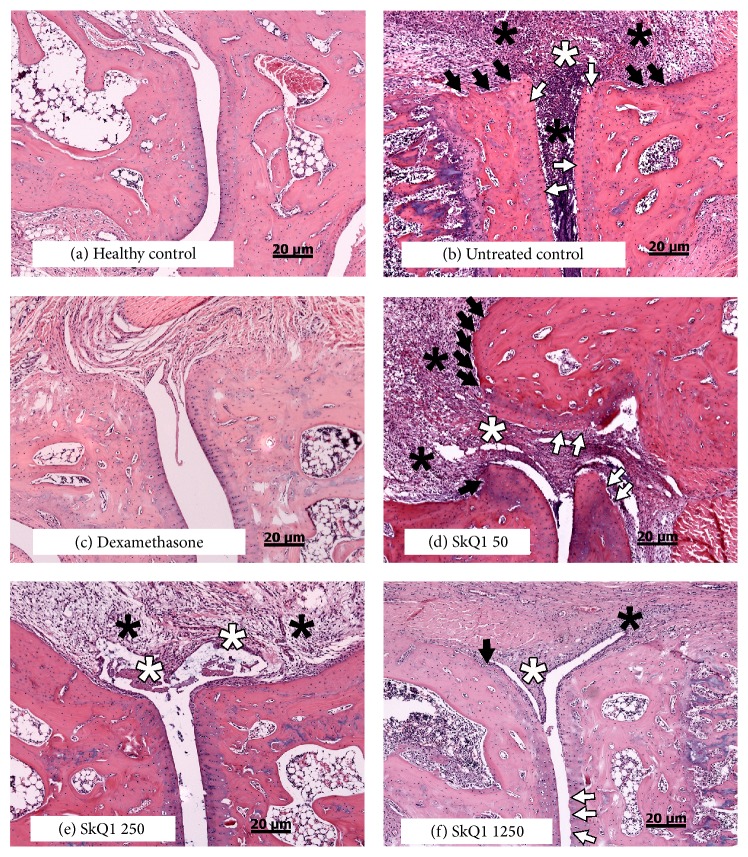
Microscopy of typical pathological lesions of paw joints of treated and untreated rats with experimental rheumatoid arthritis in experiment II. Animals were sacrificed 30 days after arthritis induction. H&E staining, 100x. (a) Normal paw joint, scores: inflammatory infiltration: 0, synovial hyperplasia: 0, cartilage destruction: 0, and bone destruction: 0; (b) untreated control: inflammatory infiltration: 3 (severe inflammatory infiltration), synovial hyperplasia: 3 (massive synovial cells proliferation and in some cases also severe destruction of synovial membrane), cartilage destruction: 2 (necrosis of cartilages), and bone destruction: 3 (numerous resorption lacunae); (c) dexamethasone: inflammatory infiltration: 0, synovial hyperplasia: 0, cartilage destruction: 0, and bone destruction: 0; (d) 50 nmol/kg/day SkQ1: inflammatory infiltration: 3, synovial hyperplasia: 3, cartilage destruction: 3 (destruction of cartilages with formation of pannus), and bone destruction: 3; (e) 250 nmol/kg/day SkQ1: inflammatory infiltration: 2 (moderate inflammatory infiltration), synovial hyperplasia: 2 (foci with more than two layers of cells in synovial membrane), cartilage destruction: 0, and bone destruction: 0; (f) 1250 nmol/kg/day SkQ1: inflammatory infiltration: 1 (mild inflammatory infiltration), synovial hyperplasia: 1 (foci with more than one layer of cells in synovial membrane), cartilage destruction: 1 (erosive surface of cartilages), and bone destruction: 1 (rare resorption lacunae). Inflammatory infiltration in joint tissues and exudation in articular cavity (black asterisks), foci of cartilage destruction (white arrows), bone resorption lacunae (black arrows), and necrotic or hyperplastic synovia (white asterisks).

**Figure 5:**
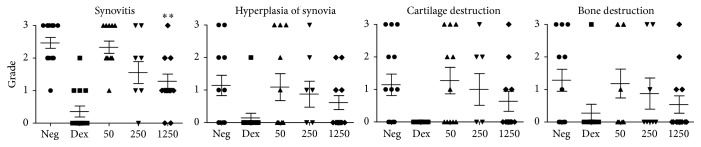
Scoring of pathological findings in SPF Wistar rats in experimental Series II that were treated with various doses of SkQ1 daily from day 1 after arthritis induction and sacrificed 30 days after arthritis induction. Typical lesions and examples of scoring are shown in Figure 4. Several fields of view and slides were examined per each animal. Statistical significance of difference between negative control and pretreated group was tested with Kruskal-Wallis test (not including dexamethasone group) with Dunn's postcorrection; ^*∗∗*^ *p* < 0.01.

Experiment II aimed to study if administration of SkQ1 into the stomach via gavage was better than administration with food. This series was carried out in a different animal facility on SPF rats obtained from another supplier and upon arthritis induction with different collagen. In this experimental series, SkQ1 administered from day 1 after arthritis induction at three doses (50, 250, and 1250 nmol/kg/day) did not have any effect on the proportion of rats that developed arthritis and only increased the mean time of disease onset from 13 to 14 days (*p* < 0.05, the Kruskal-Wallis test with Dunn's postcorrection; not significant if compared as disease-free survival in log-rank test). Pathological examination revealed a dose-dependent effect of SkQ1 on all signs of arthritis (Figure 4); however, blind scoring and statistical analysis (Figure 5) revealed that only the effect of SkQ1 on inflammatory infiltration in the group that received 1250 nm/kg/day of SkQ1 was statistically significant (ANOVA/the Kruskal-Wallis test, Dunn's postcorrection, *p* < 0.01). The effect of SkQ1 was much less pronounced than the effect of dexamethasone that was used as a positive control, but it must be noted that at this dose (1.5 mg/kg intramuscular once a week) dexamethasone produced severe weight loss in some animals.

### 3.4. Effect of SkQ1 on Neutrophil Apoptosis

**Figure 6:**
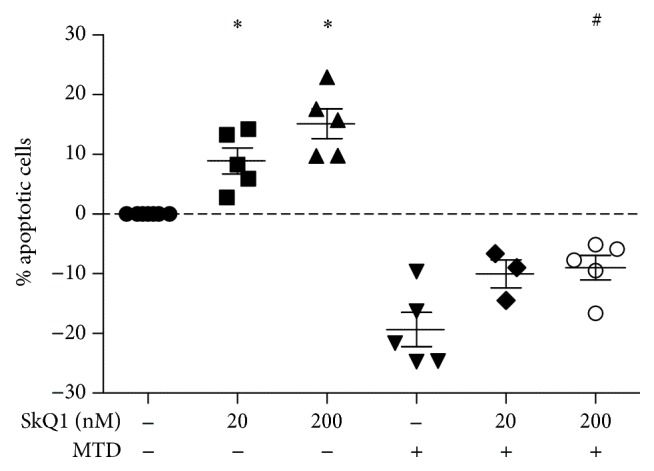
SkQ1 promotes neutrophil apoptosis in absence and in presence of a survival signal provided by mitochondrial debris (MTD). Neutrophils were incubated for 2 h in the absence or presence of 2.5 mg/mL MTD, and then SkQ1 was added at the indicated concentrations. Apoptosis was measured 22 hours after addition of SkQ1. Seven independent experiments were done using blood of different donors. Percent of apoptotic cells in individual donors varied between 49 and 75%. *y*-axis shows difference between percent of apoptotic cells in control and experimental wells. Not all tests were done for each serum, but each experimental group was reproduced 3–5 times with distinct blood samples; dots represent these distinct experiments. ANOVA with Dunn's postcorrection, ^*∗*^ *p* < 0.05 versus control; ^#^ *p* < 0.05 versus MTD-treated cells without SkQ1.

SkQ1 decreased morphological signs of arthritis even when administration was commenced late, just before the onset of overt disease. At this stage of disease, the pathogenesis is largely driven by infiltration of neutrophils that recognize cellular damage-associated molecular patterns (DAMPs). Multiple regulatory cascades acting on different levels make it hard to investigate distinct molecular pathogenesis pathways. To gain insight into the possible mechanism of SkQ1 action during the late stages of inflammation, we studied its effect on neutrophil apoptosis* in vitro*. Neutrophil apoptosis was delayed by mitochondrial DAMPs (mitochondrial debris, MTD) and promoted by SkQ1 treatment (Figure 6).

## 4. Discussion

There have been multiple lines of evidence supporting a beneficial role of antioxidants in arthritis and other autoimmune disorders. ROS have been commonly implicated in inflammation signaling (reviewed in [5]). In particular, in the model of autoimmune arthritis, overexpression of extracellular superoxide dismutase (SOD) in mice decreased disease manifestations [23], while a proteomics study implicated downregulation of redox-related proteins during pathogenesis of RA [24]. Despite this background for clinical use of antioxidants, some common antioxidants, such as vitamin E or N-acetylcysteine (NAC), were not effective in RA [10, 25]. Therefore, altering the general antioxidant status might not be sufficient for amelioration of arthritis symptoms. Interestingly, recent* in vitro* data obtained on normal human synoviocytes show that mitochondrial dysfunction induced ROS generation and inflammatory responses that could be prevented by mitochondria-targeted antioxidants [26]. In line with the theoretical predictions, SkQ1 demonstrated efficacy in reducing the key pathological signs of arthritis (inflammatory infiltration, damage to cartilage and bone) in two independent laboratories using different experimental techniques. The results of the two experimental series differed significantly. In Series I, the efficacy was very good, while in Series II the substance had an effect on infiltration by inflammatory cells but had no effect on clinical manifestations of arthritis. Importantly, the accepted clinical scoring scale takes into account mainly the paw swelling and is subject to operator mistakes, which is why the similarities in histopathology data from two series should be emphasized. Of particular importance is the fact that SkQ1 ameliorated the disruption of cartilage and bone in joints of both the paw and the knee. The absence of an effect of SkQ1 on clinical signs of inflammation in Series II is even less surprising given that similar observations (no clinical effect, but a reduction of the cartilage destruction) have been reported for another antioxidant, vitamin E [27].

Several factors could explain the difference between results in Series I and II. Models of autoimmune diseases are very sensitive to many factors, ranging from the animal food supplier to the season, and animal microbiological status constitutes a major factor. In particular, disease is more readily induced in those animals that have had less contact with diverse microflora of a conventional (non-SPF) animal facility. Therefore, we suggest that the difference in the immunological status of the rats used in Series I and Series II experiments could be one explanation for the different efficacy of SkQ1. Another major difference between the two experimental series was the administration technique. While in both series SkQ1 was given* per os*, in Series I it was given with food or into the oral cavity and in Series II it was administered into the stomach via gavage. Bioavailability of SkQ1 is significantly higher upon parenteral administration (data not shown), and, therefore, it is possible that administration of the drug into the oral cavity bypassed the limitations of intragastric administration. Therefore, a more prudent administration technique in Series II could have resulted in a lower amount of the substance being absorbed, explaining the lower apparent efficacy of SkQ1.

SkQ1 efficiently ameliorated pathological signs of experimental autoimmune arthritis in rats in both experimental series. ROS are involved in many steps of inflammation signaling, and so the potential targets of antioxidant therapy are numerous. One obvious target of mitochondrial antioxidants is the NLRP3 inflammasome [28], and, indeed, another mitochondrial rechargeable antioxidant, MitoQ, was reported to suppress activation of the NLRP3 inflammasome in a model of dextran sulfate-induced colitis [29]. This mechanism could lead to a complete prevention of arthritis in the pretreatment experiment but could hardly help in treatment (postexposure administration) protocols. However, in the model of autoimmune arthritis, there are likely additional targets of SkQ1. Administration of the compound at the day of disease onset (day 14) in Series I could partially suppress the development of arthritis. At this stage, the initial response to collagen has been long mounted, and the late-stage inflammation has commenced. One known effect of SkQ1 is the inhibition of various stages of the NF-*κ*B-signaling pathway, including I*κ*Ba phosphorylation and p65 translocation into the nucleus [30]. This pathway, induced by TNF-*α* or other stimuli, is one of the key players in RA pathogenesis [31] and therefore may be a target of SkQ1.

It should be noted that there is a controversy regarding the role of ROS in development of RA, because in certain settings NADPH oxidase (NOX2) generated ROS were shown to have a protective effect in RA [32], and abundance of reduced SH groups was correlated to increased survival of T lymphocytes and their capacity to transfer arthritis [33]. This could explain some controversial results obtained with conventional antioxidants in RA, which could have opposite effects at different stages of disease induction and at different levels of ROS metabolism. High specificity of mitochondria-targeted antioxidants can provide their beneficial role on regulation of inflammation, while avoiding potential controversial effects.

Tissue and cell injury often results in release of intracellular compounds termed damage-associated molecular patterns (DAMPs), which are recognized by the innate immune system and act as endogenous danger signals to promote and exacerbate the inflammatory response. Mitochondrial debris (MTD) have emerged as a potent source of DAMPs [34]. We have shown that the neutrophil apoptosis delay that was caused by the proinflammatory action of MTD was attenuated by SkQ1 (Figure 6). Therefore, SkQ1 may promote resolution of inflammation and prevent progressive tissue destruction. Importantly, SkQ1 prevents apoptotic and/or necrotic cell death in many cellular models at comparable concentrations [12, 21, 35]. So far, induction of apoptosis by SkQ1 at nanomolar concentrations was observed only in neutrophils. Thus, the proapoptotic effect of SkQ1 on neutrophils and the antiapoptotic effect on other cell types could hypothetically cooperate to reduce joint damage in arthritis. It is possible, however, that SkQ1 could also exert its effect on pathogenesis of experimental RA at other levels.

It is also of interest that, in experimental Series I, bone destruction was suppressed much more than the cartilage destruction. Differentiation of osteoclasts is regulated by ROS, probably via an autocrine positive-feedback loop [36], which can make them more responsive to other inflammatory stimuli by costimulating the NF-*κ*B signaling pathway [37]. Correspondingly, antioxidants have been shown to inhibit osteoclast proliferation [38, 39]. The effect of SkQ1 on osteoclast differentiation could add to its protective effect on bone destruction in the RA model.

## 5. Conclusion 

Mitochondrial antioxidant SkQ1 ameliorated inflammatory infiltration and damage to cartilage and bone in a rat model of autoimmune arthritis. These pharmacological effects were observed at a dose that is about 100 times less than the maximum tolerated dose. The magnitude of these effects does not justify use of SkQ1 as a monotherapy of rheumatoid arthritis. It is commonly acknowledged that successful treatment of RA should involve blocking of multiple inflammation signaling pathways, and SkQ1 could be a valuable supplement to the main therapy.
